# Food processing and cardiometabolic risk factors: a systematic review

**DOI:** 10.11606/s1518-8787.2020054001704

**Published:** 2020-07-20

**Authors:** Francine Silva dos Santos, Mariane da Silva Dias, Gicele Costa Mintem, Isabel Oliveira de Oliveira, Denise Petrucci Gigante

**Affiliations:** I Universidade Federal de Pelotas Faculdade de Medicina Programa de Pós-Graduação em Epidemiologia PelotasRS Brasil Universidade Federal de Pelotas. Faculdade de Medicina. Programa de Pós-Graduação em Epidemiologia. Pelotas, RS, Brasil; II Universidade Federal de Pelotas Faculdade de Nutrição Departamento de Nutrição PelotasRS Brasil Universidade Federal de Pelotas. Faculdade de Nutrição. Departamento de Nutrição. Pelotas, RS, Brasil

**Keywords:** Adult, Aged, Metabolic Syndrome, Cardiovascular Diseases, Food Consumption, Industrialized Foods, Risk factors, Nutritional Epidemiology, Systematic Review

## Abstract

**OBJECTIVE:**

To systematically review the evidence for the association between food consumption according to processing and cardiometabolic factors in adults and/or the elderly.

**METHOD:**

Two independent evaluators analyzed the electronic databases PubMed, Web of Science and Lilacs until December 2018. We used the following terms: (*convenience foods* OR *food processing* OR *highly-processed* OR *industrialized foods* OR *minimally-processed* OR *prepared foods* OR *processed foods* OR *ultra-processed* OR *ultraprocessed* OR *ultra processed* OR *unprocessed*) AND (*metabolic syndrome* OR *hypertension* OR *blood pressure* OR *diabetes mellitus* OR *glucose* OR *glycaemia* OR *insulin* OR *cholesterol* OR *triglycerides* OR *blood lipids* OR *overweight* OR *obesity*) AND (*adult* OR *adults* OR *adulthood* OR *aged* OR *elderly* OR *old*). We assessed methodological and evidence qualities, and also extracted information for the qualitative synthesis from the selected studies.

**RESULTS:**

Of the 6,423 studies identified after removing duplicates, eleven met the eligibility criteria. The main food classification we used was Nova. The consumption of ultra-processed foods was positively associated with overweight and obesity, high blood pressure and metabolic syndrome. All articles included met more than 50% of the methodological quality criteria. The quality of evidence was considered moderate for the outcome overweight and obesity and weak for hypertension and metabolic syndrome.

**CONCLUSIONS:**

The Nova food classification stands out in the area of nutritional epidemiology when assessing the effects of food processing on health outcomes. Although caution is required in the interpretation, the results indicated that the consumption of ultra-processed foods can have an unfavorable impact in the health of individuals.

## INTRODUCTION

Cardiovascular diseases (CVD) comprise the main cause of mortality in the world and approximately three quarters of deaths occur in low and middle income countries^1,2^. Risk factors for CVD include behavioral factors, such as unhealthy eating, smoking, physical inactivity and alcohol abuse^1,3^. As a consequence of behavioral risk, the most frequent cardiometabolic factors are high blood pressure (hypertension), dyslipidemia, hyperglycemia, overweight and obesity^1,3^.

Adequate and healthy feeding for a given population involves biological, environmental, social, demographic and economic aspects^4^. There are changes in eating habits worldwide, characterized by the dominance of products from the food industry^6^which are not part of the traditional food classification systems^9^. These traditional classifications are restricted to the biological properties of food, i.e., they group food according to the nutrients present in it^9^.

The monitoring of food consumption contributes to the diagnosis of the food and nutritional situation of populations and provides subsidies for the planning and organization of health services and formulation of policies and actions in the field of public health^10^. There are food classifications based on processing^8,11,12^, but there is no synthesis of evidence on the association of food consumption assessed from these classifications with the risk factors for CVD, which are a group of diseases of extreme worldwide relevance^1,3^. Thus, we intended to help elucidate the importance of such classifications in the context of nutritional epidemiology and public health and, in this study, we aimed at conducting a systematic review to assess the association between food consumption according to processing and cardiometabolic factors in adults and/or the elderly.

## METHOD

The report of this systematic review followed the Preferred Reporting Items for Systematic Reviews and Meta-Analyzes Protocol (Prisma)^13^. The study protocol was submitted to the International Prospective Register of Systematic Reviews (Prospero), being approved under number CRD42019119765.

### Search Strategy

We examined the electronic databases PubMed, Web of Science and Lilacs until December 2018. The aim was to conduct a systematic investigation of original studies that assessed the association between food consumption according to its processing and cardiometabolic factors.

To define the search terms, in addition to the Health Sciences Descriptors (DeCS) and Medical Subject Headings (MeSH), we carried out an exploratory investigation aimed at identifying keywords consistently referred to in articles in the area. Therefore, we used the following terms: (convenience foods OR food processing OR highly-processed OR industrialized foods OR minimally-processed OR prepared foods OR processed foods OR ultra-processed OR ultraprocessed OR ultra processed OR unprocessed) AND (metabolic syndrome OR hypertension OR blood pressure OR diabetes mellitus OR glucose OR glycaemia OR insulin OR cholesterol OR triglycerides OR blood lipids OR overweight OR obesity) AND (adult OR adults OR adulthood OR aged OR elderly OR old). Table 1 exemplifies the search strategy in the electronic databases. As additional research, we considered the bibliographic references of the selected articles.

**Table 1:** Search strategy in electronic databases.

Identification number	Keywords
#1	*convenience foods* OR *food processing* OR *highly-processed* OR *industrialized foods* OR *minimally-processed* OR *prepared foods* OR *processed foods* OR *ultra-processed* OR *ultraprocessed* OR *ultra processed* OR *unprocessed*
#2	*metabolic syndrome* OR *hypertension* OR *blood pressure* OR *diabetes mellitus* OR *glucose* OR *glycaemia* OR *insulin* OR *cholesterol* OR *triglycerides* OR *blood lipids* OR *overweight* OR *obesity*
#3	*adult* OR *adults* OR *adulthood* OR *aged* OR *elderly* OR *old*
	#1 AND #2 AND #3

Note: Before each set of keywords in Lilacs, “(tw:)” and in the Web of Science, “(TS=)” were included. There was a restriction for languages (English, Portuguese and Spanish) in each database.

### Eligibility Criteria

The eligible studies should present the following characteristics: I) be an original article; II) be conducted in humans; III) address the assessment of the association between food consumption according to processing (exposure) and cardiometabolic risk factors (outcome); IV) present as target population adults and/or the elderly; and V) be published in Portuguese, English or Spanish. The operationalization of the exposure allowed the inclusion of articles that used such a food classification system that considered industrial processing to define groups of foodstuffs. We excluded articles that indirectly assessed consumption, used data on availability, acquisition or commercialization of food, in addition to those whose target population comprised pregnant women or individuals with some special health condition.

### Study Selection

Two independent evaluators selected the articles to be included. Faced with cases of disagreement, a third reviewer conducted the trial. Initially, the publications were imported into the EndNote^®^ version X7 program, in which duplicates were checked, followed by reading of the titles and abstracts. The studies selected in the previous stages according to the eligibility criteria were read in full.

### Methodological Quality Assessment

We assessed the methodological quality of the selected articles according to the Strengthening the Reporting of Observational Studies in Epidemiology (Strobe)^14^ initiative, which comprises a checklist for observational studies. The maximum score to be obtained is 22 points, distributed as follows: title and/or summary (one item), introduction (two items), methodological aspects (nine items), results (five items), discussion (four items) and other information (one item – on financing)^14^. Each of the 22 items received a score of 0 or 1 considering whether it “does not meet” or “meets” each criterion, respectively. Based on the sum of the checklist’s score we established three categories for quality assessment: A, for studies that met more than 80% of the criteria; B, for studies that achieved 50 to 80% of the criteria were considered; and C, for those that met less than 50% of the criteria^15^.

### Evidence Quality Assessment

We conducted the quality of evidence for the relationship between consumption of ultra-processed foods (UPF) and each outcome by using the Grading of Recommendations Assessment, Development and Evaluation (Grade) system^16,17^. Thus, the classification of the studies was carried out as follows: A) high evidence; B) moderate evidence; C) low evidence; and D) very low evidence^16,17^. Observational studies start with low quality of evidence (C) and, among the factors that increase the level of classification, are included the magnitude of the effect, dose-response gradient and plausible confounders that may reduce the demonstrated effect or increase an unobserved effect. Among the aspects that may decrease the level of evidence are methodological quality (risk of bias), inconsistency of results, indirect evidence, imprecision and publication bias^16,17^.

### Data analysis

In order to carry out the narrative synthesis of the characteristics as the main results and descriptive approach we extracted the following information from each selected article: name of the main author, year of publication and research data collection, country of study, design, sample size and characteristics, method used to measure exposure, adjustment variables in the analysis and main results.

## RESULTS

The search strategy identified a total of 7,216 publications in the electronic databases PubMed, Web of Science and Lilacs. Out of this total, we excluded 793 as they were duplicates, resulting in 6,423 references. After analyzing the titles and abstracts, reading them in full and applying the eligibility criteria, we selected 11 studies. There was no inclusion of articles through the additional search in the reference list of selected articles. We show the complete flowchart of the selection process in Figure.

**Figure:**
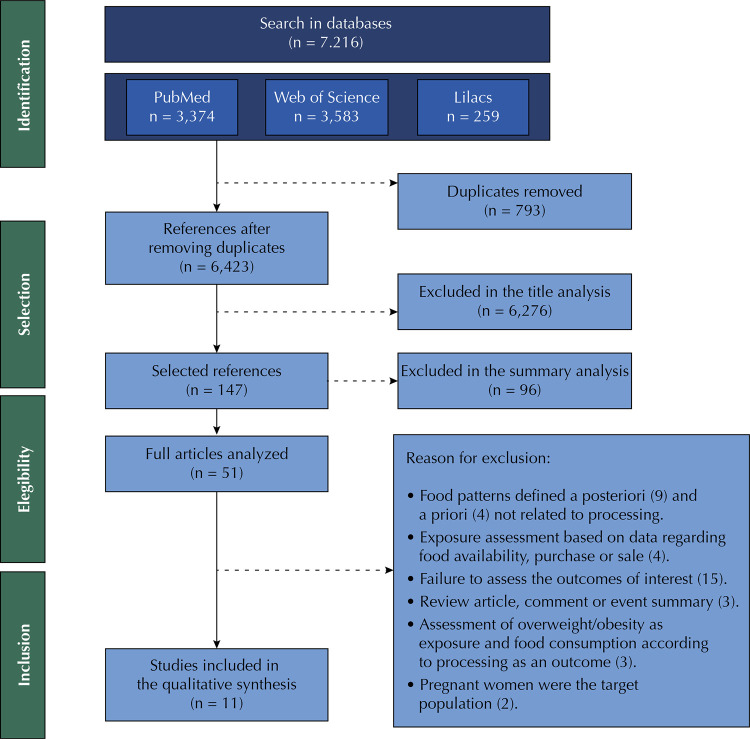
Flowchart of the selection process for the articles included in the systematic review.

### Characteristics and Methodological Quality of Included Articles

We described the characteristics and methodological quality of the publications included in this review in Table 2. Most studies (six)^18^ were conducted in high-income countries, while five publications^24^ were held in developing countries contributed, three of which carried out in Brazil^24,25,27^. As for the design, more than 80% were cross-sectional. The two cohort studies identified were conducted with the same population, which consisted of participants in *Proyecto Seguimiento Universidad de Navarra (SUN)* [University of Navarra Follow-up Project] in Spain^21,22^.

**Table 2:** Characteristics and methodological quality of the studies included in the systematic review.

Author Year of publication	Country	Design	Sample size Age range Year of research	Strobe score (%)	Study quality (Strobe)
Overweight or obesity					
Adams et al.^18^ 2015	United Kingdom	Cross-sectional	n = 2.174^a^ ≥ 18 years 2008–2012	18.0 (81.8)	A
Louzada et al.^25^ 2015	Brazil	Cross-sectional	n = 30.243 ≥ 10 years^b^ (20 to 39, 40 to 59 and ≥ 60) 2008–2009	16.0 (72.7)	B
Zhou et al.^28^ 2015	China	Cross-sectional^c^	n = 14.976 ≥ 02 years^b^ (19 to 59 and ≥ 60) 2011	15.0 (68.2)	B
Mendonça et al.^22^ 2016^d^	Spain	Cohort	n = 8.451 Mean of age = 37.7 years Median of 8,9 years of follow-up	17.0 (77.3)	B
Da Silveira et al.^24^ 2017^d^	Brazil	Cross-sectional (convenience sample)	n = 503 (vegetarians) ≥ 16 years 2015	13.0 (59.1)	B
Juul et al.^19^ 2018	United States	Cross-sectional	n = 15.977 20 to 64 years 2005–2006 and 2013–2014	19.0 (86.4)	A
Nardocci et al.^23^ 201^8^e	Canada	Cross-sectional	n = 19.363 ≥ 18 years 2004–2005	18.0 (81.8)	A
Silva et al.^27^ 2018	Brazil	Cross-sectional^c^	n = 8.977 35 to 64 years 2008–2010	21.0 (95.5)	A
Arterial hypertension					
Mendonça et al.^21^ 2017^d^	Spain	Cohort	n = 14.790 Mean of age = 32,9 to 40,0 years Mean of 9.1 (SD = 3,9) years of follow-up	20.0 (90.9)	A
Metabolic syndrome and components					
Nasreddine et al.^26^ 2018	Lebanon	Cross-sectional	n = 302 Age ≥ 18 years 2014	20.0 (90.9)	A
Lavigne-Robichaud et al.^20^ 2018	Canada	Cross-sectional	n = 811 (indigenous) ≥ 18 years 2005–2009	16.0 (72.7)	B

SD = standard deviation; Strobe: *Strengthening the Reporting of Observational Studies in Epidemiology*

^a^ 183 missings for BMI.

^b^ Results stratified by age.

^c^ Cross-sectional analysis in a cohort study.

^d^ Self-report of weight and height.

^e^ Self-report of weight and height for approximately 37% of the sample.

All articles included in this review are recent, published in the last five years (between 2015 and 2018), and data collection had taken place between 2005 and 2015. Five studies (46%) had a sample size greater than 10,000 participants^19,21,23,25,28^and the smallest sample identified evaluated 302 individuals^26^.

According to the Strobe criteria, it is possible to consider that the methodological quality of the analyzed articles was satisfactory, with an overall average of 17.5 points (minimum score: 13^24^; maximum score: 21^27^) and none were classified with quality C. Six studies^18,19,21,23,26,27^were considered of quality A and five^20,22,24,25,28^of quality B (Table 2).

The cardiometabolic risk factors identified were overweight or obesity (eight articles^18,19,22^) and arterial hypertension (one article^21^). We included two studies^20,26^that evaluated metabolic syndrome and its components because this condition is characterized by the simultaneous presence of outcomes of interest in this review^29^. Table 3 shows, in chronological order, according to the date of publication of each article and for all dependent variables, the following characteristics of the units of analysis: definition of exposure, adjustment variables in the analysis and synthesis of the main results.

**Table 3:** Summary of studies that assessed the association between processing-based food classification systems and cardiometabolic risk factors (n = 11).

Study	Exposure	Adjustment Variables	Main results
Overweight or obesity			
Adams et al.^18^ 2015	Food diary (3 to 4 days) Nova classification: NPF, PCI, UPF and NPF + PCI (% of TEI)	Gender, occupational social class, age and percentage of energy derived from alcoholic beverages.	TEI: 28% NPF, 13% PCI and 53% UPF. Higher consumption of PCI: BMI (kg / m^2^) (β = -0.09; 95%CI -0.016 – -0.03) BMI ≥ 25.0 kg / m^2^ (OR = 0.97; 95%CI 0.96 – 0.99) BMI ≥ 30.0 kg / m^2^ (OR = 0.98; 95%CI 0.97 – 0.99) Higher consumption of PCI + AMP: BMI ≥ 25.0 kg/m^2^ (OR = 0.99; 95%CI 0.98 – 0.99 No significant association was found between the consumption of NPF and UPF with the evaluated incomes.
Louzada et al.^25^ 2015	Food record (2 non-consecutive days) Nova Classification: PFN, PF and UPF (% of TEI)	Gender, age, skin color, geographic region, urbanity, education, family income per capita, smoking, physical activity, consumption of fruits, vegetables and beans and interaction between gender and income.	TEI: 68.6% NPF and 29.6% UPF (the entire sample aged ≥ 10 years). 20 to 39 years: there was no significant association between UPF consumption and the evaluated outcomes. 1st UPF consumption quintile (reference) BMI (kg/m^2^) 40 to 59 years: 2nd (β = 0.58; 95%CI 0.09 – 1.07); 3rd (β = 0.51; 95%CI 0.02 – 1.00); 4th (β = 0.70; 95%CI 0.10 – 1.31) and 5th (β = 1.12; 95%CI 0.25 – 2.00) ≥ 60 years: 2nd and 3rd quintiles with no difference between groups; 4th (β = 1.49; 95%CI 0.24 – 2.74) and 5th (β = 1.66; 95%CI 0.12 – 3.2) BMI ≥ 30,0 kg/m^2^ ≥ 60 years: 2nd (OR = 1.65; 95%CI 1.14 – 2.38); 3rd (OR = 1.74; 95%CI 1.14 – 2.67); 4th (OR = 2.07; 95%CI 1.24 – 3.45) and 5th (OR = 2.62; 95%CI 1.22 – 5.64). No association with overweight and obesity for 20 to 39 and 40 to 59 years.
Zhou et al.^28^ 2015	24h recall (3 consecutive days) PF = packaged, frozen, canned, bagged and/or packed food (kcal)	Model 1: energy intake of other foods, gender, education, per capita household income, physical activity and sedentary hours Model 2 (instrumental variables): distance from the grocery store and nearest market and urbanity + model 1.	TEI: PF 28% and 29% for participants aged 19 to 59 years and 60 years or more, respectively. 19 to 59 years: BMI (kg/m^2^) (β = 0.34; SE = 0.10) and BMI ≥ 25.0 kg/m^2^ (OR = 1.17; SE = 0.06) ≥ 60 years: BMI (kg/m^2^) (β = 0.46; SE = 0.17) and BMI ≥ 25.0 kg / m^2^ (OR = 1.13; SE = 0.10). Model 2: for adults and the elderly, there was no significant association.
Mendonça et al.^22^ 2016	FFQ (last 12 months) New Classification: UPF (portions/day)	Gender, physical activity, hours watching television, napping, smoking, “pinching” between meals, following a special diet on the baseline, BMI on the baseline and consumption of fruits and vegetables.	1st UPF consumption quartile (reference) BMI ≥ 25.0 kg/m^2^ 2nd (HR = 1.15; 95%CI 1.01 – 1.32) 3rd (HR = 1.24; 95%CI 1.09 – 1.43) 4th (HR = 1.26; 95%CI 1.10 – 1.45)
Da Silveira et al.^24^ 2017	FFQ (weekly consumption) New Classification (UPF) and sugary drinks	Time of vegetarianism.	UPF consumption at least 3x/day: 10.1%. UPF consumption ≥ 3x/day: BMI ≥ 25.0 kg/m^2^ (16 to 59 years) or ≥ 27.0 kg/m^2^ (≥ 60 years) (OR = 2.33; 95%CI 1.36 – 4.03)
Juul et al.^19^ 2018	24h recall (one day data) New Classification: UPF (% of TEI)	Model 1: age, gender, education, race / ethnicity, family poverty rate, marital status, smoking and physical activity. Model 2: EIT (mediator or confounding factor).	TEI: UPF 56.1% 5th UPF consumption quintile versus 1st quintile (reference): BMI (kg/m^2^) (β = 1.61; 95%CI 1.11 – 2.10) BMI ≥ 25.0 kg/m^2^ (OR = 1.48; 95%CI 1.25 – 1.76) BMI ≥ 30.0 kg/m^2^ (OR = 1.53; 95%CI 1.29 – 1.81) WC (cm) (β = 4.07; 95%CI 2.94 – 5.19) WC ≥ 88 or 102 cm for M and W, respectively (OR = 1.62; 95%CI 1.39 – 1.89) P-value of linear trend <0.0001 for all associations. Adjustment for energy intake did not significantly modify the associations (data not shown in the article). Interaction between gender and the relative contribution of UPF to BMI, WC and overweight.
Nardocci et al.^23^ 2018	24h recall (day before the interview) New Classification: UPF (% of TEI)	Model 1: gender, age, education and income per household. Model 2: model 1 + physical activity and smoking. Model 3: model 2 + immigrant status. Model 4: model 3 + area of residence. Model 5: model 4 + measured weight and height versus self-reported.	TEI: UPF 45% 10 p.p. increase in UPF consumption: BMI ≥ 25.0 to 29.9 kg / m^2^ (OR = 1.03; 95%CI 1.02 – 1.09). BMI ≥ 30.0 kg / m^2^ (OR = 1.05; 95%CI 1.02 – 1.08) 1st UPF consumption quartile (reference): BMI ≥ 30.0 kg / m^2^ 4th (OR = 1.32; 95%CI 1.05 – 1.57)
Silva et al.^27^ 2018	FFQ (last 12 months) New Classification: UPF (% of TEI)	Model 1: gender, age, skin color, family income per capita. Model 2: model 1 + physical activity, smoking, SAH and DM. Model 3: model 2 + energy intake of the NPF and PCI group in Nova. Model 4: model 3 + EIT.	TEI: 22.7% UPF 1st UPF consumption quartile (reference): 4th (β = 0.64; 95%CI 0.33 – 0.95) – BMI (kg / m^2^) 4th (β = 0.95; 95%CI 0.17 – 1.74) – WC (cm) 1st UPF and BMI consumption quartile <25.0 kg / m^2^ (reference): 4th (OR = 1.31; 95%CI 1.13 – 1.51) – BMI 25.0 to 29.9 kg / m^2^ 4th (OR = 1.41; 95%CI 1.18 – 1.69) – BMI ≥ 30.0 kg / m^2^ 1st quartile of UPF and WC consumption <94 cm (H) and <80 cm (M) (reference): 4th (OR = 1.41; 95%CI 1.20 – 1.66) – WC ≥ 88 or 102 cm for H and M, respectively. There was no association for category with WC ≥ 94 and <102 cm (H) and ≥ 80 and <88 cm (M).
arterial hypertension			
Mendonça et al.^21^ 2017	FFQ (last 12 months) Nova Classification: UPF (portions/day)	Model 1: gender, physical activity, hours watching television, BMI at baseline, smoking, use of analgesics, following a special diet at baseline, family history of hypertension, alcohol consumption, hypercholesterolemia. Model 2: model 1 + EIT, olive oil and fruit and vegetable intake.	3rd tertile of UPF consumption versus 1st (reference): SAH (HR = 1.21; 95%CI 1.06 – 1.37) When excluding from the TEI model (possible mediator) (HR = 1.21; 95%CI 1.07 – 1.37).
Metabolic syndrome and components			
Nasreddine et al.^26^ 2018	FFQ (last 12 months) Based on the Nova rating: two identified food patterns (ultra-processed and minimally processed/processed)	Model 1: gender, age, marital status, area of residence, education, monthly income, smoking, physical activity and EIT. Model 2: model 1 + BMI.	TEI: NPF 27.10%; PCI 12.25%; PF 23.83% and UPF 36.53%. Medium / high adhesion to the minimally processed / processed pattern was protection for: hyperglycemia (OR = 0.25; 95%CI 0.07 – 0.98) low HDL cholesterol (OR = 0.17; 95%CI 0.05 – 0.60) metabolic syndrome^30^ (OR = 0.18; 95%CI 0.04 – 0.77) When adding BMI to the model, only the association with hyperglycemia was attenuated and was not statistically significant. Ultra-processed pattern was not associated with metabolic syndrome and any of its components.
Lavigne-Robichaud et al.^20^ 2018	24-hour recall NOVA Classification (UPF), AHEI-2010 and FQS For Nova, the higher the consumption, the lower the quality of the diet. For AHEI-2010 and FQ, the higher the consumption, the better the quality of the diet.	Age, gender, area of residence, total daily energy intake, smoking and consumption of alcoholic beverages.	TEI: mean UPF of 51.9% (SD = 22.9). 5th UPF consumption quintile versus 1st quintile (reference): low HDL cholesterol (OR = 2.05; 95%CI 1.25 – 3.38) metabolic syndrome30 (OR = 1.90; 95%CI 1.14 – 3.17) UPF consumption was not associated with other components of the metabolic syndrome.

AHEI-2010: Alternative Healthy Eating Index 2010; NPF: non-processed food (*in natura*) or minimally processed; PF: processed food; UPF: ultra-processed food; WC: waist circumference; DM: diabetes mellitus; SD: standard deviation; SE: standard error; FQS: *Food Quality Score*; M: man; SAH: Systemic Arterial Hypertension; HR: Cox regression hazard ratio; 95%CI: 95% confidence interval; PCI: Processed culinary ingredients; TEI: total energy intake; BMI: Body mass index; W: women; FFQ: food frequency questionnaire; p.p.: percentual points; OR: odds ratio of logistic regression; β: coefficient of linear regression.

We identified four different methods for assessing exposure in this work, the most frequent being the food frequency questionnaire (FFQ)^21,22,24,26,27^ and the 24-hour recall^19,20,23,28^, used in five and four publications, respectively. Research was also identified that evaluated exposure through the food diary^18^ and food record^25^. The Nova classification was present in nine studies to define food according to processing^18^. Another article was based on Nova to determine the food groups, then carrying out an exploratory factor analysis, which identified two dietary patterns: “minimally processed/processed food pattern” and “ultra-processed food pattern”^26^. Finally, one publication defined packaged, frozen, canned, bagged and/or packed foods as processed^28^. We highlight that the association between UPF consumption and the identified health outcomes were assessed by all surveys that used the Nova classification, representing ten of the eleven reviewed surveys.

Although there was some homogeneity in food classification according to the extent and purpose of processing, the operationalization of the exposure was different among the articles analyzed. The main form of assessment was the percentage of total energy intake of the interest groups in each study, which was analyzed on a continuous basis^18,23,28^ and/or categorized into quartiles or quintiles^19,20,23,25,27^. There were articles that considered the number of daily servings^21,22^, the consumption greater than or equal to three times a day^24^ or based on Nova system to identify eating patterns^26^ *a posteriori.*

Regarding the possible confounders of the association of interest, only one article did not adjust for socioeconomic and demographic factors, as well as for any behavioral variables of cardiovascular risk, such as physical activity, smoking and/or alcohol consumption^24^. Another five studies^20,22,25,26,28^, although they controlled for socioeconomic, demographic and behavioral confounders, provided estimates only after adjustment for possible mediators, such as energy intake and consumption of other food groups.

### Overweight or Obesity

Eight publications investigated food consumption according to processing and overweight or obesity^18,19,22^. Most studies included more than one way of defining the outcome, but the adiposity measures considered only anthropometric indicators, evaluating the body mass index (BMI) as a continuous variable^18,19,25,27,28^or categorized: BMI = 25.0 to 29.9 kg / m^2^ considered overweight^23,27^, BMI ≥ 30.0 kg / m^2^ considered obesity^18,19,23,25,27^or BMI ≥ 25,0 kg/m^2^ considered overweight (includes overweight and obesity) ^18,19,22,24,25,28^. A survey considered BMI ≥ 27.0 kg/m^2^ as being overweight only for participants aged 60 or over^24^ and two articles would also assess abdominal obesity by measuring waist circumference (WC), defined as greater than or equal to 88 cm for women and 102 cm for men^19,27^.

Seven studies found a positive association among the consumption of UPF with, at least, one of the different methodologies for the operationalization of BMI^19,22^ and abdominal obesity^19,27^. In addition, four articles reported a dose-response gradient for this association^19,22,23,27^, i.e., the higher the consumption category of UPF, the higher the BMI averages^19,27^ and WC^19,27^ and the higher the risk of overweight^27^, obesity^19,23,27^, overweight^19,22^ or abdominal obesity^19,27^. Only one study did not observe a statistically significant relationship between UPF consumption and adiposity measures^18^. However, the higher consumption of processed culinary ingredients (PCI) or the combination of them with minimally processed foods (MPF) provided protection for the evaluated outcome^18^. Finally, the consumption of UPF was associated with a high BMI only among participants in the age groups from 40 to 59 years and above or equal to 60 years in a representative sample of the Brazilian population^25^.

### Arterial hypertension

Only one article evaluated arterial hypertension as the main outcome, and it was observed that the higher consumption of UPF (3rd tertile of consumption compared to 1st tertile) increased its incidence (hazard ratio [HR] = 1.21; 95%CI 1.06 –1.37) 21 (Table 3).

### Metabolic Syndrome and Components

Two studies evaluated the association between the exposure of interest in this review and metabolic syndrome^20,26^. Table 3 presents an article in which the target population included Canadian Indians that presented positive association between UPF consumption and the outcome (odds ratio [OR] = 1.90; 95%CI 1.14–3.17 )^20^. However, another study with Lebanese adults did not find a significant relationship between the “ultra-processed foods pattern” and the metabolic syndrome (OR = 1.11; 95%CI 0.26–4.65)^27^. The highest consumption of the “minimally processed/processed pattern” was a protective factor (OR = 0.18; 95%CI 0.04–0.77)^26^ for this outcome. In analyzes that considered the presence of each component of the metabolic syndrome, it was found that the consumption of UPF increases the risk of low HDL cholesterol^20^, while consumption of the “minimally processed/processed foods pattern” reduces the chance for low HDL cholesterol and hyperglycemia^26^.

In addition to the heterogeneity of the target population and the definition of exposure, there was also a difference in sample size and methods for collecting consumption data, which were obtained through the 24-hour recall^20^ and the FFQ^26^, respectively. The same reference was used in both studies to define metabolic syndrome^29^.

### Quality of Evidence

Table 4 shows the analysis of the quality of evidence according to the Grade^16,17^system. All included studies were observational and started from a low level of evidence (C). As this is a systematic review article, the evidence is indirect, and the possibility of publication bias is not ruled out; large effect magnitudes have not been identified; considerable methodological limitations were not verified in the reviewed articles, which, for the most part, were adjusted for plausible confounding factors; and the sample sizes allowed precision of the results. For the positive association between UPF consumption and overweight or obesity, a dose-response gradient with consistent results was reported in four studies; thus, the classification was raised to a moderate level of evidence (B). For the relationship between UPF consumption and the outcomes of hypertension and metabolic syndrome, the level of evidence remained low (C).

**Table 4:** Quality of evidence of the association between consumption of ultra-processed foods and cardiometabolic risk factors in adults and the elderly

Outcome	Positive association	No association	Quality of evidence (Grade)^a^
Overweight or obesity			Positive association ⨁⨁⨁◯
Mean of BMI (kg/m^2^)	Louzada et al.^25^ (2015)^b^ Silva et al.^27^ (2018) Juul et al.^19^ (2018)	Adams et al.^18^ (2015) Louzada et al.^25^ (2015)^c^	
BMI 25.0 to 29.9 kg/m^2^ (overweight)	Nardocci et al.^23^ (2018) Silva et al.^27^ (2018)	-	
BMI ≥ 25.0 kg/m^2^ (excessive weight)	Mendonça et al.^22^ (2016) Da Silveira et al.^24^ (2017)^e^ Juul et al.^19^ (2018)	Adams et al.^18^ (2015) Louzada et al.^25^ (2015)	
BMI ≥ 30.0 kg/m^2^ (obesity)	Louzada et al.^25^ (2015)^d^ Juul et al.^19^ (2018) Nardocci et al.^23^ (2018) Silva et al.^27^ (2018)	Adams et al.^18^ (2015)	
Mean of WC ≥ 88 cm (W) and 102 cm (M)	Juul et al.^19^ (2018) Silva et al.^27^ (2018)	-	
Arterial hypertension			Positive association ⨁⨁◯◯
	Mendonça et al.^21^ (2017)	-	
Metabolic syndrome			Positive association ⨁⨁◯◯
	Lavigne-Robichaud et al.^20^ (2018)	Nasreddine et al.^26^ (2018)	

WC: waist circumference; Grade: Grading of Recommendations Assessment, Development and Evaluation; M: men BMI: body mass index; W: women.

^a^ ⨁⨁⨁◯ = moderate quality of evidence (B); ⨁⨁◯◯ = low quality of evidence (C).

^b^ Age range from 40 to 59 and ≥ 60 years.

^c^ Age range from 20 to 39 years.

^d^ Age range from ≥ 60 years.

^e^ BMI ≥ 27.0 kg/m^2^ (≥ 60 years).

Note: Zhou et al. (2015)^29^ was not included in this assessment because exposure was processed foods. No study showed a negative association with the outcomes of interest.

## DISCUSSION

This systematic review identified and summarized the results of 11 studies that assessed the association between food consumption according to processing and cardiometabolic factors in adults and/or the elderly. Three outcomes were verified: overweight or obesity, arterial hypertension and metabolic syndrome. According to the reviewed articles, the level of evidence was considered moderate for the first outcome and low for the other evaluated morbidities.

This is our first systematic knowledge review in which we proposed to assess food consumption according to cardiometabolic processing and outcomes, considering the food classifications defined initially. It is important to highlight that this review did not restrict the search for a food classification. However, especially regarding the UPF group, Nova was used in ten out of the eleven studies included, possibly due to its international recognition and validity in the field of public health and nutritional epidemiology^8,9,30,31^. Nova considers nutritional and non-nutritional attributes of foods that can influence eating behavior, nutritional quality of food and health outcomes^19^. We emphasize that Brazil was a pioneer in using Nova to support national guidelines for food and nutrition^4^.

The unfavorable nutritional profile related to the consumption of UPF, which has an impact on the nutritional quality of food^32^, possibly stimulates the execution of research that evaluates the repercussion of this consumption on negative health outcomes. Thus, the inference of biological plausibility is feasible, considering that the high intake of UPF characterizes a diet with higher concentrations of sodium, sugar, total and saturated fats, with reduced fiber and protein content, highly energetic^18,33^, presenting high glycemic index^38^ that promotes inflammatory processes resulting from changes in the composition and metabolism of the intestinal microbiota, favoring metabolic disorders^39^. Two other articles found protective effect on the consumption of minimally processed and processed foods by people who are overweight^18^, and those presenting metabolic syndrome and some of its components (hyperglycemia and low HDL cholesterol)^26^.

The available evidence for overweight or obesity was much more abundant in relation to the other dependent variables as identified in eight studies^19,22^. The only research that did not report positive association between UPF consumption and the referred outcome analyzed together two groups from Nova (processed and ultra-processed foods)^18^, which may have contributed to such result. After the period stipulated for inclusion of articles in this review, the results of a randomized clinical trial with a crossover methodology were released, which verified an increase in body weight and energy intake of the participants during the two weeks they maintained the diet with consumption of UPF^40^. Thus, the positive association reported by this review with moderate level of evidence corroborates the results of a recent clinical trial^40^, a type of design that can raise the level of evidence^16,17^. It is worth mentioning that an association in the same direction was found in two^41,42^of three cross-sectional articles^34,41,42^which were excluded from this review because they analyzed anthropometric measures of adiposity as exposure to the consumption of UPF. Previous studies of narrative review on UPF consumption and obesity^43^ and systematic review on UPF consumption and body adiposity during childhood and adolescence^44^ reinforce the results that foods belonging to this group can contribute to increase body adiposity.

For arterial hypertension and metabolic syndrome, one^21^ and two^20,26^articles respectively, were identified. The three surveys pointed out that the consumption of UPF increased the risk for the evaluated outcomes^20,21,26^. The level of evidence was low, given that there are not enough studies to guarantee confidence in the results. In one of the articles^26^ presenting metabolic syndrome as outcome, the exposure was “ultra-processed foods pattern” and, although Nova was used to define the food groups, exploratory factor analysis added foods that do not belong to the UPF in this pattern, which led to the conclusion that the effect may have been diluted, with a confidence interval that included the unit^26^.

Due to the heterogeneity in the operationalization of the exposure and outcome variables, it was impossible to perform a quantitative synthesis (meta-analysis). This fact introduces a limitation in summarizing these results. As a limitation of this review, we highlight that all articles included presented observational design. We also highlight that experimental studies are justified in the face of considerable observational evidence^45^, and the scarcity of this type of design can be explained by the recent publication dates of the observational studies we identified. In addition, the logistical difficulties of the time between exposure and the incidence of outcomes, added to the ethical reasons that contribute to limit the amount of experimental references that assess the impact of food on health in humans^45^ need to be considered. The greater difficulty in publishing studies with negative results or without association and the language restriction in the selection of articles^17^ could lead to publication bias, since the search was carried out in indexed electronic databases. However, there is no knowledge of negative results among Brazilian researchers who develop their work in this area of knowledge.

The studies included are mostly cross-sectional, which does not allow attributing causality to the results found, and the limitations inherent to nutritional epidemiology still need to be considered, given the complexity of human nutrition and the difficulty of knowing exactly the real food consumption of individuals^45^. In order to ensure greater consistency of results, only surveys that assessed food consumption at the individual level were eligible. Finally, we emphasize that the main limitation reported in the studies included in this review was related to the collection of exposure data, as the instruments were not designed to obtain consumption information according to the extent and purpose of food processing. Considering that this is a non-differential classification error, it is possible that the results of the studies have underestimated the magnitudes of association.

As for the five studies^20,22,25,26,28^that presented only controlled estimates for possible mediators and considering the approaches used, this adjustment may lead to an underestimation in the association measure or introduce collision bias in the presence of confounders between the measurer and the outcome46. The association between UPF consumption and cardiometabolic factors should not differ between populations in the biological sense. However, it should be noted that socioeconomic and behavioral variables are important confounding factors. Regarding to the extrapolation of results, there was information from middle and upper income countries for the outcomes of overweight or obesity, from studies with different epidemiological designs, which showed consistency in the different populations evaluated. Such facts allow greater confidence in the generalization of the results for countries with diverse socioeconomic and behavioral characteristics, in addition to reducing the possibility that this association is due to chance or residual confusion. For the other outcomes (hypertension and metabolic syndrome), generalization should be prudent. In addition, we highlight that our proposal was to review articles whose target population consisted of adults and/or the elderly. Among the articles that included both age groups, only two^25,28^reported the number of participants in each group. Thus, we believe that most of the data comes from adult individuals, suggesting that the conclusions would be more appropriate for this age group.

In order to raise the level of evidence and guarantee the temporality and consistency of the results in different confounding scenarios, we recommend the conduction of other studies that, while maintaining the satisfactory methodological quality identified in the articles included in this review, would present longitudinal designs. We also suggest other research designs investigating the consequences of exposure to other Nova food groups.

This review allow us to conclude that the Nova food classification stands out in the area of nutritional epidemiology, which has evaluated the role of food processing and health outcomes, with UPF being more widely studied in relation to other food groups that integrate the classification. The results presented in this review allow us to suppose that the consumption of UPF can have an unfavorable impact on the health of individuals, especially contributing to increase the BMI. Considering the knowledge that diet entails a cardiovascular risk factor that can be modified and that the outcomes assessed in the reviewed studies comprised cardiometabolic factors^1^, in addition to the already described impact described of UPF on cardiovascular disease mortality in the United Kingdom^47^ and Brazil^48^, this study may contribute to strengthening scientific evidence that underlies public policies related to the area of food and nutrition and the coping with cardiovascular diseases. In order to reduce the population’s consumption of UPF, Brazil has shown important advances^4^, but there are still several challenges to be achieved nationally and internationally^49^.

## References

[1] 1. Word Health Organization. Technical package for cardiovascular disease management in primary health care: healthy-lifestyle counselling. Geneva: WHO; 2018 [cited 2019 Apr 25]. Available from: http://www.who.int/iris/handle/10665/260422

[2] 2. Thomas H, Diamond J, Vieco A, Chaudhuri S, Shinnar E, Cromer S, et al. Global Atlas of Cardiovascular Disease 2000-2016: the path to prevention and control. Global Heart. 2018;13(3):143-63. 10.1016/j.gheart.2018.09.511 30301680

[3] 3. World Health Organization. Global action plan for the prevention and control of noncommunicable diseases 2013-2020. Geneva: WHO; 2013 [cited 2019 Apr 25]. Available from: https://www.who.int/nmh/publications/ncd-action-plan/en/

[4] 4. Ministério da Saúde (BR), Secretaria de Atenção à Saúde, Departamento de Atenção Básica. Guia alimentar para a população brasileira. Brasília, DF; 2014 [cited 2019 Apr 25]. Available from: https://bvsms.saude.gov.br/bvs/publicacoes/guia_alimentar_populacao_brasileira_2ed.pdf

[5] 5. Lock K, Smith RD, Dangour AD, Keogh-Brown M, Pigatto G, Hawkes C, et al. Health, agricultural, and economic effects of adoption of healthy diet recommendations. Lancet. 2010;376(9753):1699-709. 10.1016/S0140-6736(10)61352-9 21074259

[6] 6. Popkin BM. The nutrition transition and its health implications in lower-income countries. Public Health Nutr. 1998;1(1):5-21. 10.1079/PHN19980004 10555527

[7] 7. Moodie R, Stuckler D, Monteiro C, Sheron N, Neal B, Thamarangsi T, et al. Profits and pandemics: prevention of harmful effects of tobacco, alcohol, and ultra-processed food and drink industries. Lancet. 2013;381(9867):670-9. 10.1016/s0140-6736(12)62089-3 23410611

[8] 8. Moubarac JC, Parra DC, Cannon G, Monteiro CA. Food classification systems based on food processing: significance and implications for policies and actions: a systematic literature review and assessment. Curr Obes Rep. 2014;3(2):256-72. 10.1007/s13679-014-0092-0 26626606

[9] 9. Monteiro CA, Cannon G, Moubarac JC, Levy RB, Louzada MLC, Jaime PC. The UN Decade of Nutrition, the NOVA food classification and the trouble with ultra-processing. Public Health Nutr. 2018;21(1):5-17. 10.1017/S1368980017000234 PMC10261019 28322183

[10] 10. Ministério da Saúde (BR), Secretaria de Atenção à Saúde. Departamento de Atenção Básica. Orientações para avaliação de marcadores de consumo alimentar na atenção básica. Brasília, DF; 2015 [cited 2019 Apr 25]. Available from: http://bvsms.saude.gov.br/bvs/publicacoes/marcadores_consumo_alimentar_atencao_basica.pdf

[11] 11. Sartori AGO, Silva MV. Main food sources of energy, nutrients and dietary fiber, according to the purpose and degree of processing, for beneficiary adolescents of the ‘Bolsa Família’ Program in Brazil. Food Public Health. 2014;4(3):151-61. 10.5923/j.fph.20140403.10

[12] 12. Poti JM, Mendez MA, Ng SW, Popkin BM. Is the degree of food processing and convenience linked with the nutritional quality of foods purchased by US households? Am J Clin Nutr. 2015;101(6):1251-62. 10.3945/ajcn.114.100925 PMC4441809 25948666

[13] 13. Moher D, Shamseer L, Clarke M, Ghersi D, Liberati A, Petticrew M, et al. Preferred Reporting Items for Systematic Review and Meta-Analysis Protocols (PRISMA-P) 2015 statement. Syst Rev. 2015;4(1):1. 10.1186/2046-4053-4-1 PMC4320440 25554246

[14] 14. Elm E, Altman DG, Egger M, Pocock SJ, Gøtzsche PC, Vandenbroucke JP. The Strengthening the Reporting of Observational Studies in Epidemiology (STROBE) statement: guidelines for reporting observational studies. Lancet. 2007;370(9596):1453-7. 10.1016/s0140-6736(07)61602-x 18064739

[15] 15. Silva DFO, Lyra CO, Lima SCVC. Padrões alimentares de adolescentes e associação com fatores de risco cardiovascular: uma revisão sistemática. Cienc Saude Coletiva. 2016;21(4):1181-96. 10.1590/1413-81232015214.08742015 27076017

[16] 16. Guyatt G, Oxman AD, Akl EA, Kunz R, Vist G, Brozek J, et al. GRADE guidelines: 1. Introduction - GRADE evidence profiles and summary of findings tables. J Clin Epidemiol. 2011;64(4):383-94. 10.1016/j.jclinepi.2010.04.026 21195583

[17] 17. Schünemann H BJ, Guyatt G, Oxman A, editors. GRADE handbook for grading quality of evidence and strength of recommendation approach. GRADE Working Group; 2010. Disponível em: https://gdt.gradepro.org/app/handbook/handbook.html

[18] 18. Adams J, White M. Characterisation of UK diets according to degree of food processing and associations with socio-demographics and obesity: cross-sectional analysis of UK National Diet and Nutrition Survey (2008-12). Int J Behav Nutr Phys Act. 2015;12:160. 10.1186/s12966-015-0317-y PMC4683717 26684833

[19] 19. Juul F, Martinez-Steele E, Parekh N, Monteiro CA, Chang VW. Ultra-processed food consumption and excess weight among US adults. Br J Nutr. 2018;1120(1):90-100. 10.1017/S0007114518001046 29729673

[20] 20. Lavigne-Robichaud M, Moubarac JC, Lantagne-Lopez S, Johnson-Down L, Batal M, Laouan Sidi EA, et al. Diet quality indices in relation to metabolic syndrome in an Indigenous Cree (Eeyouch) population in northern Québec, Canada. Public Health Nutr. 2018;21(1):172-80. 10.1017/s136898001700115x PMC10260753 28683844

[21] 21. Mendonça RD, Lopes ACS, Pimenta AM, Gea A, Martinez-Gonzalez MA, Bes-Rastrollo M. Ultra-processed food consumption and the incidence of hypertension in a Mediterranean cohort: The Seguimiento Universidad de Navarra Project. Am J Hypertens. 2017;30(4):358-66. 10.1093/ajh/hpw137 27927627

[22] 22. Mendonça RD, Pimenta AM, Gea A, Fuente-Arrillaga C, Martinez-Gonzalez MA, Lopes AC, et al. Ultraprocessed food consumption and risk of overweight and obesity: the University of Navarra Follow-Up (SUN) cohort study. Am J Clin Nutr. 2016;104(5):1433-40. 10.3945/ajcn.116.135004 27733404

[23] 23. Nardocci M, Leclerc BS, Louzada ML, Monteiro CA, Batal M, Moubarac JC. Consumption of ultra-processed foods and obesity in Canada. Can J Public Health. 2019;110(1):4-14. 10.17269/s41997-018-0130-x PMC6964616 30238324

[24] 24. Silveira JAC, Meneses SS, Quintana PT, Santos VS. Association between overweight and consumption of ultra-processed food and sugar-sweetened beverages among vegetarians. Rev Nutr. 2017;30(4):431-41. 10.1590/1678-98652017000400003

[25] 25. Louzada MLC, Baraldi LG, Steele EM, Martins APB, Canella DS, Moubarac JC, et al. Consumption of ultra-processed foods and obesity in Brazilian adolescents and adults. Prev Med. 2015;81:9-15. 10.1016/j.ypmed.2015.07.018 26231112

[26] 26. Nasreddine L, Tamim H, Itani L, Nasrallah MP, Isma’eel H, Nakhoul NF, et al. A minimally processed dietary pattern is associated with lower odds of metabolic syndrome among Lebanese adults. Public Health Nutr. 2018;21(1):160-71. 10.1017/s1368980017002130 PMC5729841 28965534

[27] 27. Silva FM, Giatti L, Figueiredo RC, Molina MCB, Cardoso LO, Duncan BB, et al. Consumption of ultra-processed food and obesity: cross sectional results from the Brazilian Longitudinal Study of Adult Health (ELSA-Brasil) cohort (2008-2010). Public Health Nutr. 2018;21(12):2271-9. 10.1017/s1368980018000861 PMC11106008 29642958

[28] 28. Zhou Y, Du S, Su C, Zhang B, Wang H, Popkin BM. The food retail revolution in China and its association with diet and health. Food Policy. 2015;55:92-100. 10.1016/j.foodpol.2015.07.001 PMC4513366 26217068

[29] 29. Alberti KGMM, Eckel RH, Grundy SM, Zimmet PZ, Cleeman JI, Donato KA, et al. Harmonizing the metabolic syndrome: a joint interim statement of the International Diabetes Federation Task Force on Epidemiology and Prevention; National Heart, Lung, and Blood Institute; American Heart Association; World Heart Federation; International Atherosclerosis Society; and International Association for the Study of Obesity. Circulation. 2009;120(16):1640-5. 10.1161/CIRCULATIONAHA.109.192644 19805654

[30] 30. Food and Agriculture Organization of the United Nations. Guidelines on the collection of information on food processing through food consumption surveys. Rome: FAO; 2015 [cited 2019 Apr 25]. Available from: http://www.fao.org/3/a-i4690e.pdf

[31] 31. Pan American Health Organization. Ultra-processed food and drink products in Latin America: trends, impact on obesity, policy implications. Washington, DC: PAHO; 2015 [cited 2019 Apr 25]. Available from: http://iris.paho.org/xmlui/handle/123456789/7699

[32] 32. Louzada MLC, Canella DS, Jaime PC, Monteiro CA. Alimentação e saúde: a fundamentação científica do guia alimentar para a população brasileira. São Paulo: Faculdade de Saúde Pública da Universidade de São Paulo; 2019. 10.11606/9788588848344

[33] 33. Louzada MLC, Martins APB, Canella DS, Baraldi LG, Levy RB, Claro RM, et al. Alimentos ultraprocessados e perfil nutricional da dieta no Brasil. Rev Saude Publica. 2015;49:38. 10.1590/S0034-8910.2015049006132

[34] 34. Bielemann RM, Motta JV, Minten GC, Horta BL, Gigante DP. Consumption of ultra-processed foods and their impact on the diet of young adults. Rev Saude Publica. 2015;49:28. 10.1590/S0034-8910.2015049005572 PMC4560335 26018785

[35] 35. Louzada ML, Martins AP, Canella DS, Baraldi LG, Levy RB, Claro RM, et al. Impact of ultra-processed foods on micronutrient content in the Brazilian diet. Rev Saude Publica. 2015;49:45. 10.1590/S0034-8910.2015049006211 PMC4560336 26270019

[36] 36. Martinez Steele E, Raubenheimer D, Simpson SJ, Baraldi LG, Monteiro CA. Ultra-processed foods, protein leverage and energy intake in the USA. Public Health Nutr. 2018;21 Spec Nº:114-24. 10.1017/S1368980017001574 PMC10260799 29032787

[37] 37. Rauber F, Louzada MLC, Steele EM, Millett C, Monteiro CA, Levy RB. Ultra-processed food consumption and chronic non-communicable diseases-related dietary nutrient profile in the UK (2008-2014). Nutrients. 2018;10(5):587. 10.3390/nu10050587 PMC5986467 29747447

[38] 38. Fardet A. Minimally processed foods are more satiating and less hyperglycemic than ultra-processed foods: a preliminary study with 98 ready-to-eat foods. Food Funct. 2016;7(5):2338-46. 10.1039/c6fo00107f 27125637

[39] 39. Zinöker MK, Lindseth IA. The Western diet-microbiome-host interaction and its role in metabolic disease. Nutrients. 2018;10(3):365. 10.3390/nu10030365 PMC5872783 29562591

[40] 40. Hall KD, Ayuketah A, Brychta R, Walter PJ, Yang S, Zhou M, et al. Clinical and Translational Report Ultra-Processed Diets Cause Excess Calorie Intake and Weight Gain: An Inpatient Randomized Controlled Trial of Ad Libitum Food Intake. Cell Metab. 2019;30(1):1–11. 10.1016/j.cmet.2019.05.008 PMC7959109 31269427

[41] 41. Djupegot IL, Nenseth CB, Bere E, Bjornara HBT, Helland SH, Overby NC, et al. The association between time scarcity, sociodemographic correlates and consumption of ultra-processed foods among parents in Norway: a cross-sectional study. BMC Public Health. 2017;17:447. 10.1186/s12889-017-4408-3 PMC5433068 28506318

[42] 42. Julia C, Martinez L, Alles B, Touvier M, Hercberg S, Mejean C, et al. Contribution of ultra-processed foods in the diet of adults from the French NutriNet-Santé study. Public Health Nutr. 2018;21 Spec Nº 1:27-37. 10.1017/s1368980017001367 PMC10260813 28703085

[43] 43. Poti JM, Braga B, Qin B. Ultra-processed food intake and obesity: what really matters for health-processing or nutrient content? Curr Obes Rep. 2017;6(4):420-31. 10.1007/s13679-017-0285-4 PMC5787353 29071481

[44] 44. Costa CS, Del-Ponte B, Assunção MCF, Santos IS. Consumption of ultra-processed foods and body fat during childhood and adolescence: a systematic review. Public Health Nutr. 2018;21 Spec N 1:148-59. 10.1017/S1368980017001331 PMC10260745 28676132

[45] 45. Willett WC. Nutritional epidemiology. In: Rothman KJ, Greenland S, Lash TL. Modern epidemiology. 3. ed. Philadelphia, PA: Lippincott-Raven; 2008. p. 1188- 205.

[46] 46. Richiardi L, Bellocco R, Zugna D. Mediation analysis in epidemiology: methods, interpretation and bias. Int J Epidemiol. 2013;42(5):1511-9. 10.1093/ije/dyt127 24019424

[47] 47. Moreira PVL, Baraldi LG, Moubarac JC, Monteiro CA, Newton A, Capewell S, et al. Comparing different policy scenarios to reduce the consumption of ultra-processed foods in UK: impact on cardiovascular disease mortality using a modelling approach. PLoS One. 2015;10(2):e0118353. 10.1371/journal.pone.0118353 PMC4334511 25679527

[48] 48. Moreira PVI, Hyseni L, Moubarac JC, Martins APB, Baraldi LG, Capewell S, et al. Effects of reducing processed culinary ingredients and ultra-processed foods in the Brazilian diet: a cardiovascular modelling study. Public Health Nutr. 2018;21(1):181-8. 10.1017/S1368980017002063 PMC10260804 28885137

[49] 49. Monteiro CA, Cannon GJ. The role of the transnational ultra-processed food industry in the pandemic of obesity and its associated diseases: problems and solutions. World Nutr. 2019;10(1):89-99. 10.26596/wn.201910189-99

