# Supplementary material for: Food processing and cardiometabolic risk factors: a systematic review
Source: Rev Saude Publica. 2020 Jul 20;54:70. doi: 10.11606/s1518-8787.2020054001704 (PMC7371411; doi:10.11606/s1518-8787.2020054001704)
Supplement: Translation

# Translation: Processamento de alimentos e fatores de risco cardiometabólicos: revisão sistemática

**Authors:** Francine Silva dos Santos, Mariane da Silva Dias, Gicele Costa Mintem, Isabel Oliveira de Oliveira, Denise Petrucci Gigante

**Affiliations:** I Brasil Universidade Federal de Pelotas. Faculdade de Medicina. Programa de Pós-Graduação em Epidemiologia. Pelotas, RS, Brasil; II Brasil Universidade Federal de Pelotas. Faculdade de Nutrição. Departamento de Nutrição. Pelotas, RS, Brasil

**Keywords:** Adulto, Idoso, Síndrome Metabólica, Doenças Cardiovasculares, Consumo de Alimentos, Alimentos Industrializados, Fatores de Risco, Epidemiologia Nutricional, Revisão Sistemática

## Abstract

**OBJETIVO:**

Revisar sistematicamente as evidências da associação entre consumo de alimentos de acordo com o processamento e fatores cardiometabólicos em adultos e idosos.

**MÉTODOS:**

Dois avaliadores independentes analisaram as bases de dados eletrônicas PubMed, *Web of Science* e Lilacs até dezembro de 2018. Os seguintes termos foram utilizados: (*convenience foods* OR *food processing* OR *highly-processed* OR *industrialized foods* OR *minimally-processed* OR *prepared foods* OR *processed foods* OR *ultra-processed* OR *ultraprocessed* OR *ultra processed* OR *unprocessed*) AND (*metabolic syndrome* OR *hypertension* OR *blood pressure* OR *diabetes mellitus* OR *glucose* OR *glycaemia* OR *insulin* OR *cholesterol* OR *triglycerides* OR *blood lipids* OR *overweight* OR *obesity*) AND (*adult* OR *adults* OR *adulthood* OR *aged* OR *elderly* OR *old*). Nos estudos incluídos foram avaliadas as qualidades metodológica e de evidência, além de extraídas informações para a síntese qualitativa.

**RESULTADOS:**

Dos 6.423 estudos identificados após a remoção das duplicatas, onze preencheram os critérios de elegibilidade. A principal classificação de alimentos utilizada foi a Nova. O consumo de alimentos ultraprocessados foi positivamente associado com excesso de peso e obesidade, hipertensão arterial e síndrome metabólica. Todos os artigos incluídos preencheram mais de 50% dos critérios de qualidade metodológica. A qualidade de evidência foi considerada moderada para o desfecho excesso de peso e obesidade e fraca para hipertensão arterial e síndrome metabólica.

**CONCLUSÕES:**

A classificação de alimentos Nova se destaca na área da epidemiologia nutricional ao avaliar os efeitos do processamento de alimentos sobre desfechos em saúde. Embora seja necessária prudência na interpretação, os resultados indicam que o consumo de alimentos ultraprocessados pode ter impacto desfavorável sobre a saúde dos indivíduos.

## INTRODUÇÃO

As doenças cardiovasculares (DCV) compreendem a principal causa de mortalidade no mundo e aproximadamente três quartos dos óbitos ocorrem em países de baixa e média renda^1,2^. Os fatores de risco para DCV incluem os comportamentais, como alimentação não saudável, tabagismo, inatividade física e uso abusivo de álcool^1,3^. Como consequência do risco comportamental, os fatores cardiometabólicos mais frequentes são pressão arterial elevada (hipertensão), dislipidemias, hiperglicemia, excesso de peso e obesidade^1,3^.

A alimentação adequada e saudável de uma população envolve aspectos biológicos, ambientais, sociais, demográficos e econômicos^4^. Observam-se alterações dos hábitos alimentares em nível mundial, caracterizadas pelo domínio de produtos provenientes da indústria alimentícia^6^ e que não fazem parte dos sistemas de classificação de alimentos tradicionais^9^. Essas classificações tradicionais se restringem às propriedades biológicas da alimentação, ou seja, agrupam os alimentos em conformidade com os nutrientes neles presentes^9^.

O monitoramento do consumo alimentar colabora para execução do diagnóstico da situação alimentar e nutricional das populações e fornece subsídios para o planejamento e a organização dos serviços de saúde e a formulação de políticas e ações no âmbito da saúde pública^10^. Existem classificações de alimentos fundamentadas no processamento^8,11,12^, mas não há uma síntese de evidências sobre a associação do consumo alimentar avaliado a partir dessas classificações e os fatores de risco para DCV, as quais são um grupo de enfermidades de extrema relevância mundial^1,3^. Dessa forma, pretende-se também contribuir para elucidar a importância dessas classificações no âmbito da epidemiologia nutricional e da saúde pública. Assim, o objetivo deste estudo foi realizar uma revisão sistemática sobre o consumo de alimentos de acordo com o processamento e identificar a associação com fatores cardiometabólicos em adultos e idosos.

## MÉTODOS

O relato desta revisão sistemática seguiu o protocolo do *Preferred Reporting Items for Systematic Reviews and Meta-Analyses* (Prisma)^13^. O protocolo de estudo foi submetido ao *International Prospective Register of Systematic Reviews* (Prospero), sendo aprovado sob o número CRD42019119765.

### Estratégia de Busca

As bases de dados eletrônicas PubMed, *Web of Science* e Lilacs foram examinadas até dezembro de 2018. O intuito foi realizar uma investigação sistemática de estudos originais que avaliaram a associação entre o consumo de alimentos de acordo com o processamento e os fatores cardiometabólicos.

Para definir os termos de busca, além dos Descritores em Ciências da Saúde (DeCS) e *Medical Subject Headings* (MeSH), foi realizada uma investigação exploratória com o propósito de identificar palavras-chave consistentemente referidas em artigos da área. Sendo assim, foram utilizados os seguintes termos: (*convenience foods* OR *food processing* OR *highly-processed* OR *industrialized foods* OR *minimally-processed* OR *prepared foods* OR *processed foods* OR *ultra-processed* OR *ultraprocessed* OR *ultra processed* OR *unprocessed*) AND (*metabolic syndrome* OR *hypertension* OR *blood pressure* OR *diabetes mellitus* OR *glucose* OR *glycaemia* OR *insulin* OR *cholesterol* OR *triglycerides* OR *blood lipids* OR *overweight* OR *obesity*) AND (*adult* OR *adults* OR *adulthood* OR *aged* OR *elderly* OR *old*). A Tabela 1 exemplifica a estratégia de busca nas bases de dados eletrônicas. Como pesquisa adicional, foram consideradas as referências bibliográficas dos artigos selecionados.

**Tabela 1:** Estratégia de busca nas bases de dados eletrônicas.

Número de identificação	Palavras-chave
#1	*convenience foods* OR *food processing* OR *highly-processed* OR *industrialized foods* OR *minimally-processed* OR *prepared foods* OR *processed foods* OR *ultra-processed* OR *ultraprocessed* OR *ultra processed* OR *unprocessed*
#2	*metabolic syndrome* OR *hypertension* OR *blood pressure* OR *diabetes mellitus* OR *glucose* OR *glycaemia* OR *insulin* OR *cholesterol* OR *triglycerides* OR *blood lipids* OR *overweight* OR *obesity*
#3	*adult* OR *adults* OR *adulthood* OR *aged* OR *elderly* OR *old*
	#1 AND #2 AND #3

Nota: Antes de cada conjunto de palavras-chave na Lilacs, foi incluído “(tw:)” e na Web of Science, “(TS=)”. Houve restrição para idiomas (inglês, português e espanhol) em cada base de dados.

### Critérios de Elegibilidade

Os estudos elegíveis deveriam apresentar os seguintes aspectos: I) ser artigo original; II) ser conduzido em humanos; III) tratar da avaliação da associação entre o consumo de alimentos de acordo com o processamento (exposição) e os fatores de risco cardiometabólicos (desfecho); IV) apresentar como população-alvo adultos e/ou idosos; e V) ser publicado em português, inglês ou espanhol. A operacionalização da exposição permitiu incluir artigos que utilizaram um sistema de classificação de alimentos que considera o processamento industrial para definição dos grupos de gêneros alimentícios. Artigos que avaliaram o consumo de forma indireta, por meio de dados de disponibilidade, aquisição ou comercialização de alimentos, além daqueles cuja população-alvo compreendia gestantes ou indivíduos com alguma condição de saúde especial, foram excluídos.

### Seleção dos Estudos

Dois avaliadores independentes realizaram a seleção dos artigos a serem incluídos. Diante de casos de discordância, um terceiro revisor conduziu o julgamento. Inicialmente as publicações foram importadas para o programa EndNote^®^ versão X7, no qual foi conduzida a verificação de duplicatas, seguida da leitura dos títulos e dos resumos. Os estudos selecionados nas etapas anteriores de acordo com os critérios de elegibilidade foram lidos na íntegra.

### Avaliação da Qualidade Metodológica

A qualidade metodológica dos artigos selecionados foi avaliada de acordo com a iniciativa *Strengthening the Reporting of Observational Studies in Epidemiology* (Strobe)^14^, que compreende uma lista de verificação para estudos observacionais. A pontuação máxima a ser obtida é de 22 pontos, distribuídos da seguinte forma: título e/ou resumo (um item), introdução (dois itens), aspectos metodológicos (nove itens), resultados (cinco itens), discussão (quatro itens) e outras informações (um item – sobre financiamento)^14^. Cada um dos 22 itens recebeu a pontuação de 0 a 1 ao considerar se “não atende” ou “atende” a cada critério, respectivamente. Com base na soma dessa pontuação, foram estabelecidas três categorias para avaliação da qualidade: A, para os estudos que preencheram mais de 80% dos critérios; B, quando foram contemplados de 50 a 80% dos critérios; e C, quando menos de 50% dos critérios foram preenchidos^15^.

### Avaliação da Qualidade de Evidência

A qualidade de evidência para a relação entre consumo de alimentos ultraprocessados (AUP) e cada desfecho foi conduzida utilizando o sistema *Grading of Recommendations Assessment, Development and Evaluation* (Grade)^16,17^. Assim, a classificação dos estudos foi realizada da seguinte forma: A) evidência alta; B) evidência moderada; C) evidência baixa; e D) evidência muito baixa^16,17^. Os estudos observacionais se iniciam com qualidade de evidência baixa (C) e, entre os fatores que elevam o nível de classificação, estão inclusos a presença de magnitude do efeito, de gradiente de dose-resposta e de confundidores plausíveis que possam reduzir o efeito demonstrado ou aumentar um efeito não observado. Entre os aspectos que podem diminuir o nível de evidência se incluem a qualidade metodológica (risco de viés), inconsistência dos resultados, evidências indiretas, imprecisão e viés de publicação^16,17^.

### Análise dos Dados

A fim de realizar a síntese narrativa das características como principais resultados e abordagem descritiva, as seguintes informações foram extraídas de cada artigo selecionado: nome do autor principal, ano de publicação e de coleta dos dados da pesquisa, país de realização do estudo, delineamento, caracterização e tamanho da amostra, método utilizado para mensurar a exposição, variáveis de ajuste na análise e principais resultados.

## RESULTADOS

A estratégia de busca identificou um total de 7.216 publicações nas bases de dados eletrônicas PubMed, *Web of Science* e Lilacs. Dessas, 793 eram duplicatas, as quais foram excluídas, resultando em 6.423 referências. Após a análise dos títulos e resumos, leitura na íntegra e aplicação dos critérios de elegibilidade, 11 estudos foram selecionados. Não houve inclusão de artigos por meio da busca adicional na lista de referências dos artigos selecionados. O fluxograma completo do processo de seleção é apresentado na Figura 1.

**Figura 1:** Fluxograma do processo de seleção dos artigos incluídos na revisão sistemática.

### Características e Qualidade Metodológica dos Estudos Incluídos

As características e a qualidade metodológica das publicações incluídas na presente revisão são descritas na Tabela 2. A maioria dos estudos (seis)^18^ foi conduzida em países de renda alta, enquanto países em desenvolvimento contribuíram com cinco publicações^24^, três delas realizadas no Brasil^24,25,27^. Quanto ao delineamento, mais de 80% eram transversais. Os dois estudos de coorte identificados foram realizados com a mesma população, que consistia de participantes do projeto de Seguimento da Universidade de Navarra (SUN) na Espanha^21, 22^.

**Tabela 2:** Características e qualidade metodológica dos estudos incluídos na revisão sistemática.

Autor Ano de publicação	País	Delineamento	Tamanho da amostra Faixa etária Ano de realização da pesquisa	PontuaçãoStrobe (%)	Qualidade do estudo (Strobe)
Excesso de peso ou obesidade					
Adams et al.^18^ 2015	Reino Unido	Transversal	n = 2.174^a^ ≥ 18 anos 2008–2012	18,0 (81,8)	A
Louzada et al.^25^ 2015	Brasil	Transversal	n = 30.243 ≥ 10 anos^b^ (20 a 39, 40 a 59 e ≥ 60) 2008–2009	16,0 (72,7)	B
Zhou et al.^28^ 2015	China	Transversal^c^	n = 14.976 ≥ 02 anos^b^ (19 a 59 e ≥ 60) 2011	15,0 (68,2)	B
Mendonca et al.^22^ 2016^d^	Espanha	Coorte	n = 8.451 Média de idade = 37,7 anos Mediana de 8,9 anos de acompanhamento	17,0 (77,3)	B
Da Silveira et al.^24^ 2017^d^	Brasil	Transversal (amostra de conveniência)	n = 503 (vegetarianos) ≥ 16 anos 2015	13,0 (59,1)	B
Juul et al.^19^ 2018	Estados Unidos	Transversal	n = 15.977 20 a 64 anos 2005–2006 e 2013–2014	19,0 (86,4)	A
Nardocci et al.^23^ 201^8^e	Canadá	Transversal	n = 19.363 ≥ 18 anos 2004–2005	18,0 (81,8)	A
Silva et al.^27^ 2018	Brasil	Transversal^c^	n = 8.977 35 a 64 anos 2008–2010	21,0 (95,5)	A
Hipertensão arterial					
Mendonca et al.^21^ 2017^d^	Espanha	Coorte	n = 14.790 Média de idade = 32,9 a 40,0 anos Média de 9,1 (DP = 3,9) anos de acompanhamento	20,0 (90,9)	A
Síndrome metabólica e componentes					
Nasreddine et al.^26^ 2018	Líbano	Transversal	n = 302 Idade ≥ 18 anos 2014	20,0 (90,9)	A
Lavigne-Robichaud et al.^20^ 2018	Canadá	Transversal	n = 811 (indígenas) ≥ 18 anos 2005–2009	16,0 (72,7)	B

DP = desvio-padrão; Strobe: *Strengthening the Reporting of Observational Studies in Epidemiology*

^a^ 183 *missings* para IMC.

^b^ Resultados estratificados por idade.

^c^ Análise transversal em um estudo de coorte.

^d^ Autorrelato de peso e altura.

^e^ Autorrelato de peso e altura para aproximadamente 37% da amostra.

Todos os artigos incluídos nesta revisão são recentes, publicados nos últimos cinco anos (entre 2015 e 2018), e a coleta de dados tinha ocorrido no período entre 2005 e 2015. Cinco estudos (46%) apresentaram um tamanho amostral superior a 10.000 participantes^19,21,23,25,28^ e a menor amostra identificada avaliou 302 indivíduos^26^.

Segundo os critérios Strobe, é possível considerar que a qualidade metodológica dos artigos analisados foi satisfatória, com uma média geral de 17,5 pontos (pontuação mínima: 13^24^; pontuação máxima: 21^27^) e nenhum foi classificado com qualidade C. Seis estudos^18,19,21,23,26,27^ foram considerados de qualidade A e cinco^20,22,24,25,28^ de qualidade B (Tabela 2).

Os fatores de risco cardiometabólico identificados foram excesso de peso ou obesidade (oito artigos^18,19,22^) e hipertensão arterial (um artigo^21^). Dois estudos^20,26^ que avaliaram síndrome metabólica e seus componentes foram incluídos, tendo em vista que essa condição é caracterizada pela presença simultânea dos desfechos de interesse desta revisão^29^. Em ordem cronológica conforme a data de publicação do artigo, para cada variável dependente são apresentadas as seguintes características das unidades de análise: definição da exposição, variáveis de ajuste na análise e síntese dos principais resultados (Tabela 3).

**Tabela 3:** Síntese dos estudos que avaliaram a associação entre sistemas de classificação de alimentos baseados no processamento e fatores de risco cardiometabólicos (n = 11).

Estudo	Exposição	Variáveis de ajuste	Principais resultados
Excesso de peso ou obesidade			
Adams et al.^18^ 2015	Diário alimentar (3 a 4 dias) Classificação Nova: AMP, ICP, AUP e AMP + ICP (% da IET)	Sexo, classe social ocupacional, idade e porcentagem de energia derivada de bebidas alcoólicas.	IET: 28% AMP, 13% ICP e 53% AUP. Maior consumo de ICP: IMC (kg/m^2^) (β = -0,09; IC95% -0,016 – -0,03) IMC ≥ 25,0 kg/m^2^ (RO = 0,97; IC95% 0,96 – 0,99) IMC ≥ 30,0 kg/m^2^ (RO = 0,98; IC95% 0,97 – 0,99) Maior consumo de ICP + AMP: IMC ≥ 25,0 kg/m^2^ (RO = 0,99; IC95% 0,98 – 0,99) Não houve associação significativa do consumo de AMP e AUP com os desfechos avaliados.
Louzada et al.^25^ 2015	Registro alimentar (2 dias não consecutivos) Classificação Nova: AMP, AP e AUP (% da IET)	Sexo, idade, cor da pele, região geográfica, urbanidade, escolaridade, renda familiar *per capita*, tabagismo, atividade física, consumo de frutas, vegetais e feijões e interação sexo e renda.	IET: 68,6% AMP e 29,6% AUP (toda a amostra com idade ≥ 10 anos). 20 a 39 anos: não houve associação significativa entre consumo de AUP e os desfechos avaliados. 1º quintil de consumo de AUP (referência) IMC (kg/m^2^) 40 a 59 anos: 2º (β = 0,58; IC95% 0,09 – 1,07); 3º (β = 0,51; IC95% 0,02 – 1,00); 4º (β = 0,70; IC95% 0,10 – 1,31) e 5º (β = 1,12; IC95% 0,25 – 2,00) ≥ 60 anos: 2º e 3º quintis sem diferença entre grupos; 4º (β = 1,49; IC95% 0,24 – 2,74) e 5º (β = 1,66; IC95% 0,12 – 3,2) IMC ≥ 30,0 kg/m^2^ ≥ 60 anos: 2º (RO = 1,65; IC95% 1,14 – 2,38); 3º (RO = 1,74; IC95% 1,14 – 2,67); 4º (RO = 2,07; IC95% 1,24 – 3,45) e 5º (RO = 2,62; IC95% 1,22 – 5,64). Sem associação com excesso de peso e obesidade para 20 a 39 e 40 a 59 anos.
Zhou et al.^28^ 2015	Recordatório de 24h (3 dias consecutivos) AP = alimentos embalados, congelados, enlatados, ensacados e/ou empacotados (kcal)	Modelo 1: ingestão energética de outros alimentos, sexo, escolaridade, renda domiciliar *per capita*, atividade física e horas sedentárias. Modelo 2 (variáveis instrumentais): distância da mercearia e mercado mais próximo e urbanidade + modelo 1.	IET: AP 28% e 29% para os participantes com 19 a 59 anos e 60 anos ou mais, respectivamente. 19 a 59 anos: IMC (kg/m^2^) (β = 0,34; EP = 0,10) e IMC ≥ 25,0 kg/m^2^ (RO = 1,17; EP = 0,06) ≥ 60 anos: IMC (kg/m^2^) (β = 0,46; EP = 0,17) e IMC ≥ 25,0 kg/m^2^ (RO = 1,13; EP = 0,10). Modelo 2: para adultos e idosos, não houve associação significativa.
Mendonca et al.^22^ 2016	QFA (últimos 12 meses) Classificação Nova: AUP (porções/dia)	Sexo, atividade física, horas assistindo televisão, sono de sesta, tabagismo, “beliscar” entre as refeições, seguir uma dieta especial no *baseline,* IMC no *baseline* e consumo de frutas e vegetais.	1º quartil de consumo de AUP (referência) IMC ≥ 25,0 kg/m^2^ 2º (HR = 1,15; IC95% 1,01 – 1,32) 3º (HR = 1,24; IC95% 1,09 – 1,43) 4º (HR = 1,26; IC95% 1,10 – 1,45)
Da Silveira et al.^24^ 2017	QFA (consumo semanal) Classificação Nova (AUP) e bebidas açucaradas	Tempo de vegetarianismo.	Consumo de AUP no mínimo 3x/dia: 10,1%. Consumo de AUP ≥ 3x/dia: IMC ≥ 25,0 kg/m^2^ (16 a 59 anos) ou ≥ 27,0 kg/m^2^ (≥ 60 anos) (RO = 2,33; IC95% 1,36 – 4,03)
Juul et al.^19^ 2018	Recordatório de 24h (dados de um dia) Classificação Nova: AUP (% da IET)	Modelo 1: idade, sexo, escolaridade, raça/etnia, taxa de pobreza familiar, estado civil, tabagismo e atividade física. Modelo 2: IET (mediador ou fator de confusão).	IET: AUP 56,1% 5º quintil de consumo de AUP *versus* 1º quintil (referência): IMC (kg/m^2^) (β = 1,61; IC95% 1,11 – 2,10) IMC ≥ 25,0 kg/m^2^ (RO = 1,48; IC95% 1,25 – 1,76) IMC ≥ 30,0 kg/m^2^ (RO = 1,53; IC95% 1,29 – 1,81) CC (cm) (β = 4,07; IC95% 2,94 – 5,19) CC ≥ 88 ou 102 cm para H e M, respectivamente (RO = 1,62; IC95% 1,39 – 1,89) Valor-p de tendência linear < 0,0001 para todas as associações. Ajuste para ingestão energética não modificou de forma significativa as associações (dados não apresentados no artigo). Interação entre sexo e contribuição relativa de AUP para IMC, CC e excesso de peso.
Nardocci et al.^23^ 2018	Recordatório de 24h (dia anterior à entrevista) Classificação Nova: AUP (% da IET)	Modelo 1: sexo, idade, escolaridade e renda por domicílio. Modelo 2: modelo 1 + atividade física e tabagismo. Modelo 3: modelo 2 + status de imigrante. Modelo 4: modelo 3 + área de residência. Modelo 5: modelo 4 + peso e altura mensurados *versus* autorrelatados.	IET: AUP 45% Aumento de 10 p.p. no consumo de AUP: IMC ≥ 25,0 a 29,9 kg/m^2^ (RO = 1,03; IC95% 1,02 – 1,09). IMC ≥ 30,0 kg/m^2^ (RO = 1,05; IC95% 1,02 – 1,08) 1º quartil de consumo de AUP (referência): IMC ≥ 30,0 kg/m^2^ 4º (RO = 1,32; IC95% 1,05 – 1,57)
Silva et al.^27^ 2018	QFA (últimos 12 meses) Classificação Nova: AUP (% da IET)	Modelo 1: sexo, idade, cor da pele, renda familiar *per capita*. Modelo 2: modelo 1 + atividade física, tabagismo, HAS e DM. Modelo 3: modelo 2 + ingestão energética do grupo de AMP e ICP da Nova. Modelo 4: modelo 3 + IET.	IET: 22,7% AUP 1º quartil de consumo de AUP (referência): 4º (β = 0,64; IC95% 0,33 – 0,95) – IMC (kg/m^2^) 4º (β = 0,95; IC95% 0,17 – 1,74) – CC (cm) 1º quartil de consumo de AUP e IMC < 25,0 kg/m^2^ (referência): 4º (RO = 1,31; IC95% 1,13 – 1,51) – IMC 25,0 a 29,9 kg/m^2^ 4º (RO = 1,41; IC95% 1,18 – 1,69) – IMC ≥ 30,0 kg/m^2^ 1º quartil de consumo de AUP e CC < 94 cm (H) e < 80 cm (M) (referência): 4º (RO = 1,41; IC95% 1,20 – 1,66) – CC ≥ 88 ou 102 cm para H e M, respectivamente.Não houve associação para categoria com CC ≥ 94 e < 102 cm (H) e ≥ 80 e < 88cm (M).
Hipertensão arterial			
Mendonca et al.^21^ 2017	QFA (últimos 12 meses) Classificação Nova: AUP (porções/dia)	Modelo 1: sexo, atividade física, horas assistindo televisão, IMC no *baseline*, tabagismo, uso de analgésicos, seguir uma dieta especial no *baseline,* história familiar de hipertensão, consumo de álcool, hipercolesterolemia. Modelo 2: modelo 1 + IET, ingestão de azeite de oliva e de frutas e vegetais.	3º tercil de consumo de AUP *versus* 1º (referência): HAS (HR = 1,21; IC95% 1,06 – 1,37) Ao excluir do modelo IET (possível mediador) (HR = 1,21; IC95% 1,07 – 1,37).
Síndrome metabólica e componentes			
Nasreddine et al.^26^ 2018	QFA (últimos 12 meses) Baseado na classificação Nova: dois padrões alimentares identificados (ultraprocessados e minimamente processado/processado)	Modelo 1: sexo, idade, situação conjugal, área de residência, escolaridade, renda mensal, tabagismo, atividade física e IET. Modelo 2: modelo 1 + IMC.	IET: AMP 27,10%; ICP 12,25%; AP 23,83% e AUP 36,53%. Adesão média/elevada ao padrão minimamente processado/processado foi proteção para: hiperglicemia (RO = 0,25; IC95% 0,07 – 0,98) baixo colesterol HDL (RO = 0,17; IC95% 0,05 – 0,60) síndrome metabólica^30^ (RO = 0,18; IC95% 0,04 – 0,77) Ao acrescentar IMC no modelo, apenas a associação com hiperglicemia foi atenuada e não foi estatisticamente significativa. Padrão ultraprocessados não foi associado com síndrome metabólica e nenhum dos seus componentes.
Lavigne-Robichaud et al.^20^ 2018	Recordatório de 24h Classificação NOVA (AUP), AHEI-2010 e FQS Para a Nova, quanto maior o consumo, menor a qualidade da dieta. Para AHEI-2010 e FQ, quanto maior o consumo, melhor a qualidade da dieta.	Idade, sexo, área de residência, ingestão energética diária total, tabagismo e consumo de bebidas alcóolicas.	IET: AUP média de 51,9% (DP = 22,9). 5º quintil de consumo de AUP *versus* 1º quintil (referência): baixo colesterol HDL (RO = 2,05; IC95% 1,25 – 3,38) síndrome metabólica^30^ (RO = 1,90; IC95% 1,14 – 3,17) Consumo de AUP não foi associado com demais componentes da síndrome metabólica.

AHEI-2010: *Alternative Healthy Eating Index 2010*; AMP: alimentos não processados (*in natura*) ou minimamente processados; AP: alimentos processados; AUP: alimentos ultraprocessados; CC: circunferência da cintura; DM: diabetes mellitus; DP: desvio-padrão; EP: erro padrão; FQS: *Food Quality Score*; H: homens; HAS: hipertensão arterial sistêmica; HR: *hazard ratio* da regressão de Cox; IC95%: intervalo de confiança de 95%; ICP: ingredientes culinários processados; IET: ingestão energética total; IMC: índice de massa corporal; M: mulheres; QFA: questionário de frequência alimentar; p.p.: pontos percentuais; RO: razão de *odds* da regressão logística; β: coeficiente da regressão linear

Este trabalho identificou quatro diferentes métodos para avaliação da exposição, os mais frequentes sendo o questionário de frequência alimentar (QFA)^21,22,24,26,27^ e o recordatório de 24 horas^19,20,23,28^, utilizados em cinco e quatro publicações, respectivamente. Também foram identificadas pesquisas que avaliaram a exposição pelo diário alimentar^18^ e registro alimentar^25^. A classificação Nova esteve presente em nove estudos para definição de alimentos de acordo com processamento^18^. Outro artigo se baseou na Nova para determinar os grupos de alimentos, executando a seguir uma análise fatorial exploratória, que identificou dois padrões alimentares: “padrão alimentos minimamente processados/processados” e “padrão alimentos ultraprocessados”^26^. Por fim, uma publicação definiu como processados os alimentos embalados, congelados, enlatados, ensacados e/ou empacotados^28^. Salienta-se que a associação entre consumo de AUP e os desfechos em saúde identificados foram avaliados por todas as pesquisas que utilizaram a classificação Nova, representando dez das onze pesquisas revisadas.

Embora tenha ocorrido certa homogeneidade na classificação dos alimentos segundo a extensão e o propósito do processamento, a operacionalização da exposição foi distinta entre os artigos analisados. A principal forma de avaliação foi o percentual da ingestão energética total dos grupos de interesse de cada estudo, sendo esse analisado na forma contínua^18,23,28^ e/ou categorizada em quartis ou quintis^19,20,23,25,27^. Houve aqueles que consideraram o número de porções diárias^21,22^, o consumo maior ou igual a três vezes por dia^24^ ou baseado na Nova para identificação de padrões alimentares *a posteriori*^26^.

Em relação aos possíveis confundidores da associação de interesse, apenas um artigo não ajustou para fatores socioeconômicos e demográficos, bem como para nenhuma variável comportamental de risco cardiovascular, tais como atividade física, tabagismo e/ou consumo de bebidas alcoólicas^24^. Outros cinco estudos^20,22,25,26,28^, embora tenham controlado para confundidores socioeconômicos, demográficos e comportamentais, forneceram apenas as estimativas após ajuste para possíveis mediadores, como a ingestão energética e o consumo de outros grupos de alimentos.

### Excesso de Peso ou Obesidade

Oito publicações investigaram o consumo de alimentos de acordo com o processamento e excesso de peso, sobrepeso ou obesidade^18,19,22^. A maior parte dos estudos incluiu mais de uma forma de definição do desfecho, porém as medidas de adiposidade consideraram apenas indicadores antropométricos, avaliando o índice de massa corporal (IMC) como uma variável contínua^18,19,25,27,28^ ou categorizada: IMC = 25,0 a 29,9 kg/m^2^ considerado sobrepeso^23,27^, IMC ≥ 30,0 kg/m^2^ considerado obesidade^18,19,23,25,27^ ou IMC ≥ 25,0 kg/m^2^ considerado excesso de peso (inclui sobrepeso e obesidade)^18,19,22,24,25,28^. Uma pesquisa considerou IMC ≥ 27,0 kg/m^2^ como excesso de peso apenas para os participantes com 60 anos ou mais^24^ e dois artigos também avaliariam obesidade abdominal pela aferição da circunferência da cintura (CC), definida como maior ou igual a 88 cm para mulheres e 102 cm para homens^19,27^.

Sete pesquisas constataram uma associação positiva entre o consumo de AUP com, no mínimo, uma das distintas metodologias de operacionalização do IMC^19,22^ e obesidade abdominal^19,27^. Ademais, quatro artigos reportaram gradiente de dose-resposta para essa associação^19,22,23,27^, ou seja, quanto maior a categoria de consumo dos AUP, maiores as médias de IMC^19,27^ e CC^19,27^ e mais elevado o risco de sobrepeso^27^, obesidade^19,23,27^, excesso de peso^19,22^ ou obesidade abdominal^19,27^. Apenas um estudo não observou uma relação estatisticamente significativa entre o consumo de AUP e as medidas de adiposidade^18^. Entretanto, o maior consumo de ingredientes culinários processados (ICP) ou a combinação deles com os alimentos minimamente processados (AMP) conferiram proteção para o desfecho avaliado^18^. Finalmente, o consumo de AUP foi associado com IMC elevado apenas entre os participantes nas faixas etárias de 40 a 59 anos e maior ou igual a 60 anos em uma amostra representativa da população brasileira^25^.

### Hipertensão Arterial

Apenas um artigo avaliou como desfecho principal a hipertensão arterial, tendo sido observado que o maior consumo de AUP (3º tercil de consumo comparado ao 1º tercil) aumenta a sua incidência (*hazard ratio* [HR] = 1,21; IC95% 1,06–1,37)^21^ (Tabela 3).

### Síndrome Metabólica e Componentes

Dois estudos avaliaram a associação entre a exposição de interesse desta revisão e a síndrome metabólica^20,26^. Conforme a Tabela 3, em um artigo cuja população-alvo incluiu indígenas canadenses, foi demonstrada uma associação positiva entre o consumo de AUP e o desfecho (razão de *odds* [RO] = 1,90; IC95% 1,14–3,17)^20^. Todavia, outra pesquisa com adultos libaneses não verificou relação significativa entre o “padrão alimentos ultraprocessados” e a síndrome metabólica (RO = 1,11; IC95% 0,26–4,65)^27^. O maior consumo do “padrão minimamente processado/processado” foi um fator de proteção (RO = 0,18; IC95% 0,04–0,77)^26^ para este desfecho. Nas análises que consideraram a presença de cada componente da síndrome metabólica, verificou-se que o consumo de AUP aumenta o risco de baixo colesterol HDL^20^, enquanto, o consumo do “padrão alimentos minimamente processados/processados” reduz a chance para baixo colesterol HDL e hiperglicemia^26^.

Além da heterogeneidade da população-alvo e da definição da exposição, houve diferença também no tamanho amostral e nos métodos para coletar os dados de consumo, os quais foram obtidos por meio do recordatório de 24 horas^20^ e do QFA^26^, respectivamente. A mesma referência foi utilizada em ambos os estudos para definição de síndrome metabólica^29^.

### Qualidade de Evidência

A Tabela 4 apresenta a análise da qualidade de evidência de acordo com o sistema Grade^16,17^. Todos os estudos incluídos foram observacionais e partiram de um nível de evidência baixo (C). Por se tratar de um artigo de revisão sistemática, as evidências são indiretas, não sendo descartada a possibilidade de viés de publicação; não foram identificadas largas magnitudes de efeito; limitações metodológicas consideráveis não foram verificadas nos artigos revisados, os quais, em sua maioria, foram ajustados para fatores plausíveis de confusão; e os tamanhos amostrais permitiram precisão dos resultados. Para a associação positiva entre consumo de AUP e excesso de peso ou obesidade, em quatro estudos foi reportado um gradiente de dose-resposta com resultados consistentes; assim, a classificação foi elevada para um nível de evidência moderado (B). Para a relação entre consumo de AUP e os desfechos de hipertensão arterial e de síndrome metabólica, o nível de evidência se manteve baixo (C).

**Tabela 4:** Qualidade de evidência da associação entre consumo de alimentos ultraprocessados e fatores de risco cardiometabólicos em adultos e idosos.

Desfecho	Associação positiva	Sem associação	Qualidade da evidência (Grade)^a^
Excesso de peso ou obesidade			Associação positiva ⨁⨁⨁◯
Média de IMC (kg/m^2^)	Louzada et al.^25^ (2015)^b^ Silva et al.^27^ (2018) Juul et al.^19^ (2018)	Adams et al.^18^ (2015) Louzada et al.^25^ (2015)^c^	
IMC 25,0 a 29,9 kg/m^2^ (sobrepeso)	Nardocci et al.^23^ (2018) Silva et al.^27^ (2018)	-	
IMC ≥ 25,0 kg/m^2^ (excesso de peso)	Mendonça et al.^22^ (2016) Da Silveira et al.^24^ (2017)^e^ Juul et al.^19^ (2018)	Adams et al.^18^ (2015) Louzada et al.^25^ (2015)	
IMC ≥ 30,0 kg/m^2^ (obesidade)	Louzada et al.^25^ (2015)^d^ Juul et al.^19^ (2018) Nardocci et al.^23^ (2018) Silva et al.^27^ (2018)	Adams et al.^18^ (2015)	
Média de CC ≥ 88 cm (M) e 102 cm (H)	Juul et al.^19^ (2018) Silva et al.^27^ (2018)	-	
Hipertensão arterial			Associação positiva ⨁⨁◯◯
	Mendonca et al.^21^ (2017)	-	
Síndrome metabólica			Associação positiva ⨁⨁◯◯
	Lavigne-Robichaud et al.^20^ (2018)	Nasreddine et al.^26^ (2018)	

CC: circunferência da cintura; Grade: *Grading of Recommendations Assessment, Development and Evaluation*; H: homens; IMC: índice de massa corporal; M: mulheres.

^a^ ⨁⨁⨁◯ = qualidade de evidência moderada (B); ⨁⨁◯◯ = qualidade de evidência baixa (C).

^b^ Faixa etária de 40 a 59 e ≥ 60 anos.

^c^ Faixa etária de 20 a 39 anos.

^d^ Faixa etária de ≥ 60 anos.

^e^ IMC ≥ 27,0 kg/m^2^ (≥ 60 anos).

Nota: Zhou et al. (2015)^29^ não foi incluído nesta avaliação, pois exposição foi alimentos processados. Nenhum estudo apresentou associação negativa com os desfechos de interesse.

## DISCUSSÃO

Esta revisão sistemática identificou e sumarizou os resultados de 11 estudos que avaliaram a associação entre o consumo de alimentos de acordo com o processamento e fatores cardiometabólicos em adultos e/ou idosos. Três desfechos foram verificados: excesso de peso ou obesidade, hipertensão arterial e síndrome metabólica. De acordo com os artigos revisados, o nível de evidência foi considerado moderado para o primeiro desfecho e baixo para as demais morbidades avaliadas.

Trata-se da primeira revisão sistemática de conhecimento dos autores em que foi proposta a avaliação do consumo de alimentos de acordo com o processamento e desfechos cardiometabólicos, considerando as classificações de alimentos definidas inicialmente. É importante salientar que esta revisão não restringiu a busca para uma determinada classificação de alimentos. Contudo, com destaque para o grupo AUP, a Nova foi utilizada em dez dos onze estudos incluídos, possivelmente devido ao seu reconhecimento internacional e validade no campo da saúde pública e da epidemiologia nutricional^8,9,30,31^. A Nova considera atributos nutricionais e não nutricionais dos alimentos que podem influenciar o comportamento alimentar, a qualidade nutricional da alimentação e os desfechos em saúde^19^. Ressalta-se que o Brasil foi pioneiro ao utilizar a Nova para respaldar as diretrizes nacionais de alimentação e nutrição^4^.

O perfil nutricional desfavorável relacionado ao consumo de AUP, o qual tem impacto na qualidade nutricional da alimentação^32^, possivelmente estimula a execução de pesquisas que avaliam a repercussão desse consumo em desfechos negativos à saúde. Assim, é factível a inferência de plausibilidade biológica, tendo em vista que a ingestão elevada de AUP caracteriza uma alimentação com maiores concentrações de sódio, açúcar, gorduras totais e saturadas, com reduzido teor de fibras e proteínas, altamente energética^18,33^, com maior índice glicêmico^38^ e que promove um processo inflamatório decorrente das alterações na composição e metabolismo da microbiota intestinal, favorecendo desordens metabólicas^39^. Outros dois artigos verificaram efeito protetor do consumo de alimentos minimamente processados e processados para excesso de peso^18^, síndrome metabólica e alguns de seus componentes (hiperglicemia e baixo colesterol HDL)^26^.

As evidências disponíveis para excesso de peso ou obesidade foram largamente mais abundantes em relação às demais variáveis dependentes identificadas, de tal modo que foram identificados oito estudos^18,19,22^. A única pesquisa que não reportou associação positiva entre consumo de AUP e o desfecho referido analisou em conjunto dois grupos da Nova (alimentos processados e ultraprocessados)^18^, o que pode ter contribuído para esse resultado. Após o período estipulado para inclusão de artigos nesta revisão, foram divulgados os resultados de um ensaio clínico randomizado com metodologia *crossover*, o qual verificou aumento de peso corporal e ingestão energética dos participantes durante as duas semanas em que mantiveram a dieta com consumo de AUP^40^. Sendo assim, a associação positiva reportada por esta revisão e com nível de evidência considerado moderado corrobora com os resultados de um recente ensaio clínico^40^, tipo de delineamento que pode elevar o nível de evidência^16,17^. Cabe citar que uma associação na mesma direção foi encontrada em dois^41,42^ de três artigos transversais^34,41,42^ que foram excluídos dessa revisão por terem analisado as medidas antropométricas de adiposidade como exposição para o consumo de AUP. Estudos prévios de revisão narrativa sobre consumo de AUP e obesidade^43^ e revisão sistemática sobre consumo de AUP e adiposidade corporal durante a infância e adolescência^44^ reforçam os resultados de que os alimentos pertencentes a esse grupo podem contribuir para elevar a adiposidade corporal.

Para hipertensão arterial e síndrome metabólica foram identificados, respectivamente, um^21^ e dois^20,26^ artigos. As três pesquisas apontaram que o consumo de AUP aumentou o risco para os desfechos avaliados^20,21,26^. O nível de evidência foi baixo, tendo em vista que não há estudos suficientes para garantir confiança nos resultados. Em um dos artigos^26^ com síndrome metabólica como desfecho, a exposição foi “padrão alimentos ultraprocessados” e, embora a Nova tenha sido utilizada para definir os grupos de alimentos, a análise fatorial exploratória acrescentou alimentos que não pertencem aos AUP nesse padrão, o que levou à conclusão de que o efeito pode ter sido diluído, com um intervalo de confiança que incluiu a unidade^26^.

Em virtude da heterogeneidade na operacionalização das variáveis de exposição e desfecho, foi inviável realizar uma síntese quantitativa (meta-análise). Tal fato introduz uma limitação na sumarização desses resultados. Ainda como limitação desta revisão, cabe ressaltar que todos os artigos incluídos apresentaram delineamento observacional. Salienta-se ainda que estudos experimentais são justificáveis diante de consideráveis evidências observacionais^45^, e a escassez desse tipo de delineamento pode ser explicada pelas datas de publicação recentes dos estudos observacionais identificados. Além disso, as dificuldades logísticas do tempo entre a exposição e a incidência dos desfechos, somadas aos motivos éticos que contribuem para limitar a quantidade de referências experimentais que avaliam a repercussão da alimentação sobre a saúde em humanos^45^ precisam ser consideradas. A maior dificuldade na publicação de estudos com resultados negativos ou sem associação e a restrição de idioma na seleção dos artigos^17^ poderiam levar ao viés de publicação, uma vez que a busca foi realizada em bases de dados eletrônicas indexadas. No entanto, não se tem conhecimento de resultados negativos entre os pesquisadores brasileiros que desenvolvem seus trabalhos nessa área do conhecimento.

Os estudos incluídos são majoritariamente de delineamento transversal, o que não permite inferir causalidade para os resultados encontrados, e ainda precisam ser consideradas as limitações inerentes à epidemiologia nutricional, dada a complexidade da alimentação humana e a dificuldade de conhecer com exatidão o real consumo alimentar dos indivíduos^45^. Com o intuito de garantir maior consistência dos resultados, foram elegíveis apenas pesquisas que avaliaram o consumo alimentar em nível individual. Por fim, cabe destacar que a principal limitação relatada nos estudos incluídos nesta revisão foi referente à coleta de dados da exposição, pois os instrumentos não foram elaborados para obter informações de consumo de acordo com a extensão e o propósito do processamento dos alimentos. Considerando que esse é um erro de classificação não diferencial, é possível que os resultados dos estudos tenham subestimado as magnitudes de associação.

Quanto aos cinco estudos^20,22,25,26,28^ que apresentaram apenas estimativas controladas para possíveis mediadores, com as abordagens utilizadas, esse ajuste pode conduzir à subestimação da medida de associação ou introduzir viés de colisão na presença de confundidores entre o medidor e o desfecho^46^. A associação entre consumo de AUP e fatores cardiometabólicos não deve diferir entre populações no sentido biológico. Porém, cabe salientar que variáveis socioeconômicas e comportamentais são importantes fatores de confusão. No que se refere à extrapolação dos resultados, houve informação proveniente de países de renda média e alta para os desfechos excesso de peso ou obesidade, através de estudos com diferentes delineamentos epidemiológicos, os quais apresentaram consistência nas distintas populações avaliadas. Tais fatos permitem maior confiança na generalização dos resultados para países com diversificadas características socioeconômicas e comportamentais, além de reduzir a possibilidade de que essa associação seja decorrente do acaso ou de confusão residual. Para os demais desfechos (hipertensão e síndrome metabólica), deve existir prudência na generalização. Ademais, ressalta-se que a proposta foi revisar artigos cuja população-alvo era constituída de adultos e/ou idosos. Dentre os artigos que incluíram ambas as faixas etárias, apenas dois^25,28^ informaram o número de participantes em cada uma delas. Acredita-se que a maioria dos dados seja de indivíduos adultos, sugerindo que as conclusões seriam mais adequadas para essa faixa etária.

A fim de elevar o nível de evidências e garantir a temporalidade e a consistência dos resultados em diferentes cenários de confundimento, seriam recomendados outros estudos que, mantendo a qualidade metodológica satisfatória identificada nos artigos incluídos nesta revisão, apresentassem delineamentos longitudinais. Outras pesquisas que investiguem as consequências da exposição aos demais grupos de alimentos da Nova também são sugeridas.

A partir desta revisão, é possível concluir que a classificação de alimentos Nova se destaca na área da epidemiologia nutricional que tem avaliado o papel do processamento de alimentos e os desfechos em saúde, sendo os AUP mais largamente estudados em relação aos demais grupos de alimentos que compõem a classificação. Os resultados apresentados nesta revisão permitem supor que o consumo de AUP pode ter impacto desfavorável à saúde dos indivíduos, sobretudo contribuindo para elevar o IMC. Tendo em vista o conhecimento de que a alimentação é um fator de risco cardiovascular passível de modificação e que os desfechos avaliados nos estudos revisados compreenderam fatores cardiometabólicos^1^, além do impacto já descrito dos AUP na mortalidade por doenças cardiovasculares no Reino Unido^47^ e no Brasil^48^, o presente estudo poderá contribuir para o fortalecimento das evidências científicas que fundamentam as políticas públicas relacionadas à área de alimentação e nutrição e ao enfrentamento das doenças cardiovasculares. No intuito de reduzir o consumo de AUP da população, o Brasil tem apresentado importantes avanços^4^, mas ainda há diversos desafios a serem alcançados nacional e internacionalmente^49^.
